# Chronic hypoxia does not cause wall thickening of intra‐acinar pulmonary supernumerary arteries

**DOI:** 10.14814/phy2.12674

**Published:** 2016-01-26

**Authors:** Kaori Oshima, Jared M. McLendon, Wiltz W. Wagner, Ivan F. McMurtry, Masahiko Oka

**Affiliations:** ^1^ Department of PharmacologyUniversity of South AlabamaMobileAlabama; ^2^ Center for Lung BiologyUniversity of South AlabamaMobileAlabama; ^3^ Department of Biochemistry and Molecular BiologyUniversity of South AlabamaMobileAlabama; ^4^ Department of Internal MedicineUniversity of South AlabamaMobileAlabama

**Keywords:** Chronic hypoxia, pulmonary supernumerary artery, vascular remodeling

## Abstract

Chronic exposure to hypoxia causes pulmonary hypertension and pulmonary arterial remodeling. Although the exact mechanisms of this remodeling are unclear, there is evidence that it is dependent on hemodynamic stress, rather than on hypoxia alone. Pulmonary supernumerary arteries experience low hemodynamic stress as a consequence of reduced perfusion due to 90° branching angles, small diameters, and “valve‐like” structures at their orifices. We investigated whether or not intra‐acinar supernumerary arteries undergo structural remodeling during the moderate pulmonary hypertension induced by chronic hypoxia. Rats were exposed to either normoxia or hypoxia for 6 weeks. The chronically hypoxic rats developed pulmonary hypertension. For both groups, pulmonary arteries were selectively filled with barium–gelatin mixture, and the wall thickness of intra‐acinar pulmonary arteries was measured in histological samples. Only thin‐walled arteries were observed in normoxic lungs. In hypertensive lungs, we found both thin‐ and thick‐walled pulmonary arteries with similar diameters. Disproportionate degrees of arterial wall thickening between parent and daughter branches were observed with supernumerary branching patterns. While parent arteries developed significant wall thickening, their supernumerary branches did not. Thus, chronic hypoxia‐induced pulmonary hypertension did not cause wall thickening of intra‐acinar pulmonary supernumerary arteries. These findings are consistent with the idea that hemodynamic stress, rather than hypoxia alone, is the cause of structural remodeling during chronic exposure to hypoxia.

## Introduction

Chronic exposure to hypoxia causes pulmonary hypertension that is accompanied by functional and structural changes of the pulmonary arteries. Functionally, pulmonary arteries develop sustained vasoconstriction (Oka et al. 2008; McMurtry et al. 2010), and structurally, they undergo medial and adventitial thickening of pulmonary arteries and muscularization of pulmonary arterioles (Stenmark et al. 2006; Naeije and Dedobbeleer 2013). Recent studies by Sheikh et al. provide new insights into the molecular mechanisms of muscularization (Sheikh et al. 2014, 2015). However, the exact causes of the remodeling, in particular, the relative roles of mechanical forces (increased pressure and shear stress) and of decreased oxygen tension are unclear. Studies using the left pulmonary artery banding technique indicate that increased hemodynamic stress, rather than a direct effect of hypoxia, is responsible for the structural changes (Rabinovitch et al. 1983; Le Cras et al. 1998).

Pulmonary arteries are marked by their juxtaposition to airways. The exception to this pattern is pulmonary supernumerary arteries that branch from parent vessels without airways at 90° with significantly smaller diameters (Elliott and Reid 1965; Shaw et al. 1999). At the orifices of these branches, sphincter‐like structures have been found in human lungs (Elliott and Reid 1965) and valve‐like structures in bovine lungs (Shaw et al. 1999). Due to these properties, the perfusion through supernumerary arteries is normally low, as demonstrated by their invisibility in angiograms and the absence of endothelial flow alignment (Elliott and Reid 1965; Lane et al. 1983; Burrowes et al. 2005). Therefore, the hemodynamic stress in these vessels is predicted to be low. Accordingly, we investigated in rats whether or not intra‐acinar supernumerary arteries undergo wall thickening during the moderate pulmonary hypertension induced by chronic hypoxia.

## Materials and Methods

### Experimental animals

All experiments were approved by the institutional animal care and use committee. The investigation conforms to the *Guide for the Care and Use of Laboratory Animals* published by the US National Institutes of Health. In this study, the histological samples from a previous work (Oka et al. 2007) were examined using independent methods. Adult, male, Sprague–Dawley rats were divided into two groups. The control group (*n* = 3) was maintained at the altitude of Denver, CO (elevation 5280 feet; PO_2_ = 120 mmHg). The chronically hypoxic group (*n* = 3) was housed in a hypobaric chamber at the simulated altitude of 18,000 feet; PO_2_ = 76 mmHg, for 6 weeks. The hypobaric chamber was flushed continuously with room air to wash out CO_2_, H_2_O, and NH_3_. The chamber was opened (10–15 min) every 2 days to clean the cages and replenish the food and water. Both groups had a 12:12‐h light–dark cycle and free access to the food and water.

### Hemodynamic measurements

After 6 weeks, the rats were anesthetized with ketamine (100 mg/kg, i.m.) and xylazine (15 mg/kg, i.m.). A catheter was passed into the pulmonary artery via jugular vein and right ventricle. The rats recovered for 48 h in room air with the catheter in place. Each was placed into a room air‐ventilated transparent chamber and given time to stabilize. Mean pulmonary arterial pressure was then measured in the conscious animals. The animals were killed with an overdose of pentobarbital sodium (100 mg/kg, i.v.), and the hearts were removed to measure right ventricular hypertrophy [Fulton Index, RV/(LV + S)].

### Morphometric analysis

The pulmonary arteries were perfused with 37°C phosphate‐buffered saline at 20 cm H_2_O, and then injected with a 60°C barium sulfate–gelatin mixture at 74 mmHg for 3 min as previously described (Rabinovitch et al. 1979; Oka et al. 2007). The lungs were fixed with 10% buffered formalin via airway instillation at 36 cm H_2_O. The lungs were embedded in paraffin, and a random 5‐*μ*m section was cut from each animal and stained with hematoxylin and eosin. The pulmonary arteries were identified by the arterial‐selective filling of the barium–gelatin mixture because the injectate cannot cross the capillaries (deMello et al. 1997; Jakkula et al. 2000). The cross‐sectional, intra‐acinar pulmonary arteries with supernumerary branches were selected in both control and chronically hypoxic lungs. To exclude longitudinal parent vessels, arteries with an aspect ratio >2 were not included. Supernumerary branches were identified by their relation to the parent arteries having a branching angle of ~90° and a diameter of <50% (Townsley 2012). These criteria excluded conventional dichotomous (equal diameter) and axial (unequal diameter) branches. Clear connections between parent arteries and supernumerary branches were established with structural and injectate continuity. The diameter and wall thickness of arteries were measured using ImageJ, after the number of pixels were calibrated according to the scale bars for each magnification. The values of both diameter and wall thickness were the average of two independent measurements. A percentage of arterial wall thickness was calculated as wall thickness/external diameter × 100. One arterial structure, likely to be a rat‐specific oblique arterial segment as previous reported (Meyrick et al. 1978), in a normal lung was excluded based on Grubbs’ test (*α* = 0.05). Measurements are expressed as means ± SEM. Comparisons between the groups were made with student *t*‐test, or one‐way ANOVA with Bonferroni's multiple comparison test, with GraphPad Prism Version 4.0. Differences were considered significant at *P *<* *0.05.

## Results

The chronically hypoxic rats developed pulmonary hypertension. Mean pulmonary arterial pressure was 44 ± 3 mmHg in hypoxic rats and 23 ± 2 mmHg in control rats (*P *<* *0.05). The Fulton index, RV/LV + S, a measure of right ventricular hypertrophy, was nearly double in the hypoxic rats compared to the control rats, 64 ± 2% versus 33 ± 1% (*P *<* *0.05).

**Figure 1:**
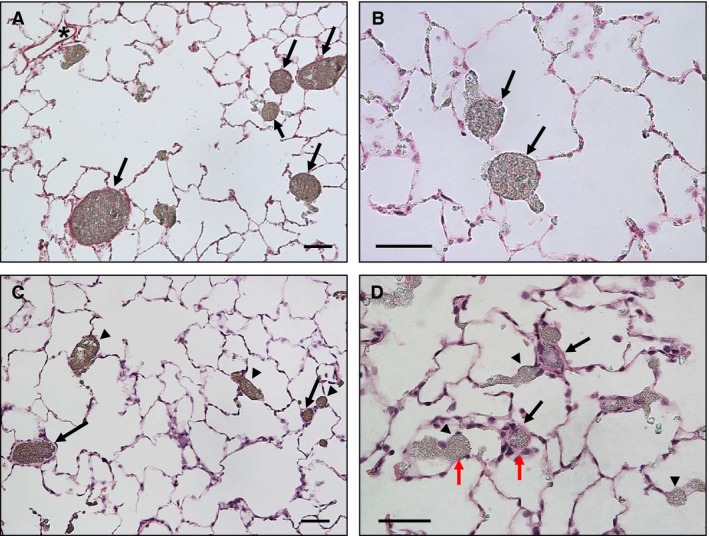
Pulmonary arteries in control and chronically hypoxic lungs. (A and B) Normal pulmonary arteries had thin walls (arrows) and pulmonary veins were not filled by barium–gelatin mixture (*). (C and D) In response to chronic hypoxia‐induced pulmonary hypertension, some of the pulmonary arteries clearly developed wall thickening (arrows), while others remained thin walled (arrowheads). Note the contrast in wall thickness in similar diameter pulmonary arteries (D, red arrows). Scale bar = 50 *μ*m.

In Figure 1A, the pulmonary arteries were readily identified as they were filled with the barium–gelatin injectate (arrows), while the pulmonary veins were unfilled (asterisk). To determine the approximate percent of thick‐ and thin‐walled arteries, five randomly selected fields (each field = 1 mm^2^) per animal were examined. The thick‐ or thin‐walled arteries were defined by comparison to normal arteries from normoxic animals, and the arteries with percent wall thickness greater than ~2% were considered thick. In control rats, the distal pulmonary arteries were consistently thin walled (Fig. 1A and B, arrows). In chronically hypoxic rats, 337 intra‐acinar pulmonary arteries (<150 *μ*m in external diameter) were found and measured. Approximately, 40% of small pulmonary arteries had significantly thickened walls (Fig. 1C and D, arrows), while ~60% of arteries in the same size range had thin walls (Fig. 1C and D, arrowheads). In Fig. 1D, the red arrows indicate two similar‐diameter arteries with or without wall thickening.

**Figure 2:**
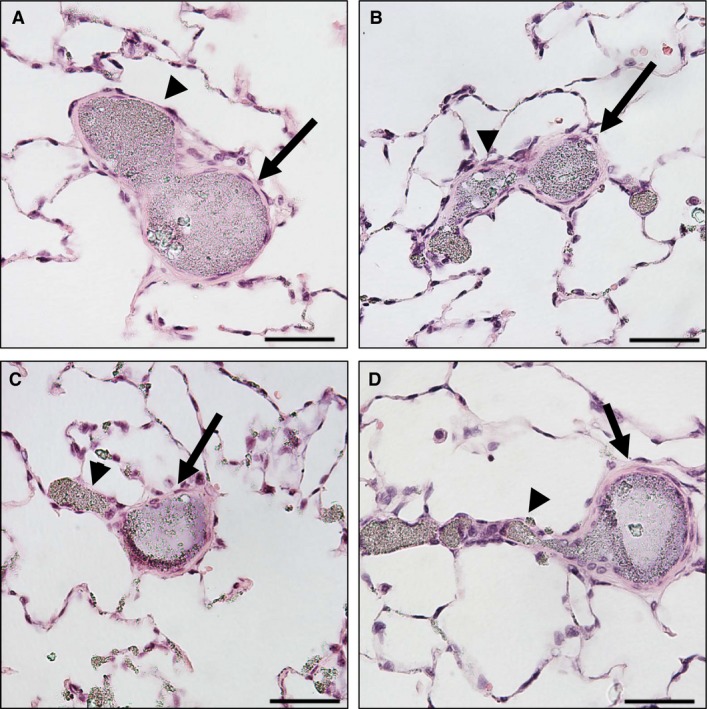
The degrees of pulmonary arterial wall thickening in relation to branching patterns in chronically hypoxic lungs. (A and B) Both parent (arrows) and daughter (arrowheads) branches developed wall thickening in response to chronic hypoxia‐induced pulmonary hypertension. The branching angles are less than 90°, suggesting they are conventional dichotomous or axial branching patterns. (C and D) Only the parent branches (arrows) developed wall thickening and the daughter, supernumerary branches remained thin walled (arrowheads). The diameters of daughter branches are considerably smaller than the diameter of parent arteries, and the branching angles are approximately 90°, which support their identification as supernumerary arteries. Scale bar = 50 *μ*m.

In chronically hypoxic lungs, conventional dichotomous and axial arterial branches developed wall thickening in both parent (Fig. 2A and B, arrows) and daughter branches (arrowheads). In contrast, supernumerary branches remained thin walled (Fig. 2C and D, arrowheads), compared to their parent arteries (arrows).

**Figure 3:**
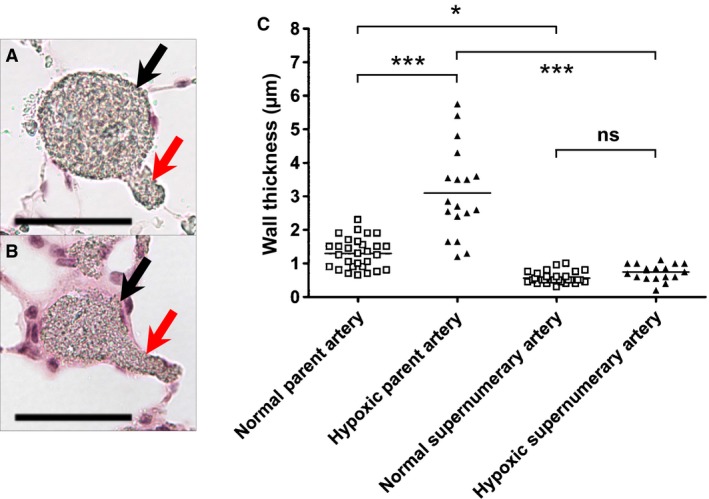
The effect of hypoxic hypertension on wall thickness of pulmonary arteries and their supernumerary branches. Wall thickness of parent arteries and daughter supernumerary branches were measured in both control and chronically hypoxic rats (*n* = 3 each). (A and B) Representative microphotographs show the measured arterial structures in control (A) and chronically hypoxic hypertensive (B) lungs. Parent arteries (black) and supernumerary branches (red) are indicated by arrows. Scale bar = 50 *μ*m. (C) Individual measurement of wall thickness of parent arteries and supernumerary branches in control and chronically hypoxic hypertensive lungs. Parent arteries developed marked wall thickening in response to hypoxia‐induced pulmonary hypertension, while supernumerary branches did not. ***P <* *0.001, *P <* *0.05, and ns = not significant by ANOVA.

The lung sections from control and chronic hypoxia groups were surveyed to identify arteries with supernumerary branches. Representative images of selected arterial structures are shown in Figure 3A (control) and Figure 3B (chronic hypoxia). In lung sections from control rats, a total of 356 possible supernumerary branches were found and 29 of those met the criteria (90° angle, <50% parent diameter, and clear connection). In lung sections from chronically hypoxic rats, a total of 240 possible supernumerary branches were found and 18 of those met the criteria. Many of the arterial structures were excluded due to unclear borders, unclear connections between parent and daughter branches, or ambiguous branching patterns. Measured wall thickness of both parent arteries and supernumerary branches are shown in Figure 3C. The wall thickness of supernumerary branches was less than half of parent arteries in both normal and chronically hypoxic lungs. In hypoxic hypertensive lungs, parent arteries developed marked wall thickness while supernumerary arteries remained thin walled (Fig. 3C).

**Figure 4:**
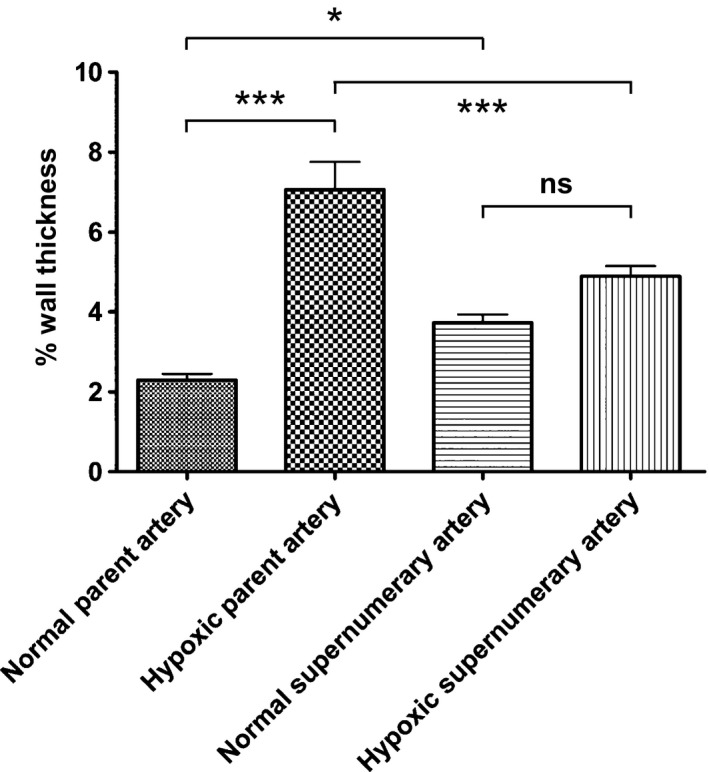
The effect of hypoxic hypertension on percent wall thickness of pulmonary arteries and their supernumerary branches. The diameter and wall thickness of parent and daughter supernumerary branches were measured in both control and hypoxic hypertensive rats (*n* = 3 each). The percent wall thickness was calculated as wall thickness/external diameter × 100. Values are mean ± SEM, ***P <* *0.001, *P <* *0.01, and ns = not significant by ANOVA.

The mean diameter of parent arteries from both control and chronic hypoxia groups was 55.8 ± 3.4 *μ*m and that of supernumerary branches was 15.6 ± 0.8 *μ*m. To account for the difference in vessel diameters between parent and supernumerary arteries, percent wall thickness was calculated, and the four groups of arteries were compared (Fig. 4). In normoxic lungs, supernumerary branches had higher percent wall thickness than parent arteries, presumably due to smaller diameter. In hypoxic hypertensive lungs, parent arteries developed significant wall thickening, while the supernumerary branches remained thin.

The sectioning angle of supernumerary branches was inevitably longitudinal, which might have affected the measurements. Therefore, we determined whether or not small pulmonary arteries that have similar diameters to supernumerary branches developed wall thickening. Cross‐sectional arteries within a diameter range from 8 to 35 *μ*m were examined in five randomly selected fields (each field = 1 mm^2^) per chronically hypoxic lung. These arteries had various degrees of wall thickening. Out of 119 vessels that were examined, 54 arteries had higher percent wall thickness (6.4 ± 0.29%) than the mean value of chronically hypoxic supernumerary branches (4.9 ± 0.26%) (*P *<* *0.05). The remainder of the population may represent small conventional pulmonary arteries that did not undergo medial thickening or unidentifiable supernumerary arteries.

## Discussion

The development of medial and adventitial thickening in pulmonary arteries during chronic exposure to hypoxia has been repeatedly shown (Hislop and Reid 1976; Rabinovitch et al. 1979; Stenmark et al. 2006). The recent reports by Sheikh et al. describe a sequence of pulmonary arterial smooth muscle progenitor cell migration, dedifferentiation, proliferation, and redifferentiation in the muscularization of arterioles (Sheikh et al. 2014). However, the exact cause and complex mechanisms of the wall thickening remain undefined (Stenmark et al. 2006). Previous studies in left pulmonary artery banded rats have shown that the cause of this structural remodeling is hemodynamic stress, rather than hypoxia alone (Rabinovitch et al. 1983; Le Cras et al. 1998). It is recognized that hemodynamic stress, such as increased mechanical forces and shear stress, is necessary for the vascular remodeling caused by chronic hypoxia (Pugliese et al. 2015). Our finding that intra‐acinar supernumerary arteries do not develop wall thickening in chronically hypoxic rats with moderate pulmonary hypertension is consistent with this idea.

The finding that not all intra‐acinar pulmonary arteries develop thick walls with chronic hypoxia is in line with a previous report (Rabinovitch et al. 1979). It is unclear what accounts for the different degrees of vascular remodeling. One possibility is that the degree of wall thickening corresponds to the degree of hemodynamic stress that each vessel experiences, depending on the branching pattern. Another possibility is the presence or absence of smooth muscle progenitor cells (Sheikh et al. 2014, 2015).

Our results have an important implication in terms of the histological quantification of pulmonary arteries and veins. The supernumerary arteries structurally resemble pulmonary veins with their lack of accompanying airways and thin walls. Particularly, in chronically hypoxic lungs, the lack of wall thickening can lead to misidentification of these arteries as veins. In order to identify these vessels in histological quantitative analyses, a clear indication of arteries, such as arterial‐specific markers or barium–gelatin injection, is required. Importantly, the frequency of supernumerary branches is greater than that of conventional branches (2–3:1) in humans (Hislop and Reid 1978). Thus, misidentification of supernumerary arteries as veins will impact studies on pulmonary vascular structures in both normal and pathological conditions.

We acknowledge limitations associated with the morphometric measurements in this study. First, the two‐dimensional morphometric analysis cannot be absolutely accurate in classification of the branching patterns, which was based on diameters and branching angles. Although we applied stringent criteria to exclude nonsupernumerary arteries, it was possible that some conventional dichotomous and axial branches were included. It was also possible that some supernumerary arteries were excluded. To define supernumerary arteries by establishing the absence of associated airways, previous studies have examined large arterial segments, namely the main axial pulmonary artery and large pulmonary arteries up to the fourth generation branch (Elliott and Reid 1965; Shaw et al. 1999). However, this was not possible with the intra‐acinar pulmonary arteries we focused on. In future studies, three‐dimensional analysis with a wider diameter range of arteries is necessary to confirm the findings and advance our understanding of these vessels. Another issue is the high pressure applied to inject the barium–gelatin mixture that might have expanded the arteries, and made the wall thickness less than that in noninjected lungs.

In conclusion, our results show that chronic hypoxia does not cause wall thickening of intra‐acinar pulmonary supernumerary arteries in rats. This is presumably because these unique vascular segments are minimally perfused and exposed to low hemodynamic stress during moderate pulmonary hypertension. It is important to further investigate the function and structure of these potential collateral pathways in severe pulmonary hypertension given their possible link to the formation of plexiform lesions (Yaginuma et al. 1990; Dickinson et al. 2013; Oshima et al. 2013). The abundance of supernumerary arteries, accounting for up to 40% of the cross‐sectional area of side branches (Elliott and Reid 1965; Hislop and Reid 1978) emphasizes the probable importance of these vessels in pulmonary physiology and pathophysiology.

## Acknowledgement

We thank Dr. Troy Stevens for the critical reading and discussion of the manuscript.

## Conflict of Interest

None declared.
